# Longevity

**Published:** 1860-01

**Authors:** 


*Longevity.*—Nothing so conduces to long life as comfortable external circumstances, which relieve the mind from those eating cares that so wear away the nervous system. Hence clergymen, who, though compelled to struggle to keep up appearances, are generally raised above actual want, are remarkable for their longevity. The most striking instance we have recently seen of the truth of this observation, is to be found in a recent number of Household Words, which informs us that, of a score or so of British Peers who have died since the commencement of the year, there were sixteen whose united ages amounted to no less than 1,229 years, giving an average of seventy-six-and-a-half years for each. A practical comment this upon the advantages of comfort.

